# Muscle strength and muscle mass phenotypes and survival in unresectable hepatocellular carcinoma receiving hepatic arterial infusion chemotherapy-based combination therapy

**DOI:** 10.3389/fonc.2026.1974237

**Published:** 2026-10-21

**Authors:** Chan Su, Guibao Ji, Xinze Wang, Qiuxia Guo, Xing Lv, Xin Jin, Ding Xiao

**Affiliations:** 1 Department of Hepatobiliary-Pancreatic and Hernia Surgery, Wuhan Fourth Hospital, Wuhan, China; 2 Department of Trauma and Orthopedics, Wuhan Fourth Hospital, Wuhan, China; 3 Department of Gastroenterology, Wuhan Fourth Hospital, Wuhan, China; 4 Hepatic Surgery Center, Tongji Hospital, Tongji Medical College, Huazhong University of Science and Technology, Wuhan, China

**Keywords:** body composition, computed tomography, handgrip strength, immune checkpoint inhibitors, prognostic stratification, skeletal muscle index

## Abstract

**Background:**

Muscle strength and muscle mass may decline discordantly in hepatocellular carcinoma (HCC), but the prognostic relevance of combined strength–mass phenotypes during hepatic arterial infusion chemotherapy (HAIC)-based combination therapy is unclear. We evaluated survival and incremental prognostic value according to handgrip strength (HGS) and skeletal muscle index (SMI) phenotypes in patients with unresectable HCC.

**Methods:**

We performed a secondary analysis of 154 patients from the 1:1 propensity score-matched subset of a previously reported 265-patient cohort receiving HAIC with targeted therapy and immunotherapy. Patients were classified as Normal, SMI-low, HGS-low, or Both-low. Progression-free survival (PFS) and overall survival (OS) were assessed using Kaplan–Meier and multivariable Cox analyses. Incremental prognostic value for OS was evaluated using Harrell’s concordance index (C-index) and likelihood-ratio tests, with internal validation using bootstrap optimism correction.

**Results:**

Median follow-up was 22.1 months, as estimated using the reverse Kaplan–Meier method. PFS and OS differed across phenotypes (both log-rank *P* < 0.001). After multivariable adjustment, HGS-low was associated with shorter PFS (hazard ratio [HR], 2.81; 95% confidence interval [CI], 1.71–4.60) and OS (HR, 3.09; 95% CI, 1.87–5.11), and Both-low showed similar associations (PFS: HR, 2.53; 95% CI, 1.40–4.56; OS: HR, 3.72; 95% CI, 2.00–6.92). The 10-patient SMI-low estimates were imprecise for PFS (HR, 0.79; 95% CI, 0.29–2.16) and OS (HR, 0.81; 95% CI, 0.28–2.31). After bootstrap optimism correction, adding low HGS increased the C-index from 0.625 to 0.705, whereas the four-phenotype model yielded 0.701 and did not improve model fit beyond low HGS (*P* = 0.798).

**Conclusion:**

Low HGS, with or without low SMI, was associated with poorer survival. HGS may provide a simple and practical measure for baseline prognostic assessment in unresectable HCC treated with HAIC-based combination therapy.

## 1 Introduction

Immune-based combinations have improved survival and changed first-line treatment for unresectable hepatocellular carcinoma (HCC) (1, 2). Outcomes nevertheless remain heterogeneous, especially in patients with extensive intrahepatic tumor burden or impaired hepatic reserve (3). In advanced HCC, modified oxaliplatin, leucovorin, and fluorouracil (mFOLFOX)-based hepatic arterial infusion chemotherapy (HAIC) has produced better tumor response and survival than sorafenib (4) and has been incorporated into combination strategies for selected patients (5, 6). Pretreatment markers that capture host vulnerability and physiological reserve may complement conventional measures of tumor burden and liver function. In advanced HCC, particularly with coexisting liver disease, systemic inflammation, reduced nutritional intake, hyperammonemia, anabolic resistance, and inactivity may contribute to muscle impairment and reduced physiological reserve (7, 8). Neuromuscular abnormalities have also been observed in cirrhosis, although their contribution to grip weakness in HCC remains uncertain (9). Muscle quantity, muscle quality, strength, and physical performance are distinct constructs (10, 11). Here, skeletal muscle index (SMI) derived from computed tomography (CT) at the third lumbar vertebral level (L3) estimated muscle quantity, whereas handgrip strength (HGS) measured grip strength rather than global muscle strength; neither muscle quality nor objective physical performance was directly assessed.

A large CT-based cohort linked low SMI with mortality across disease stages (12), while progressive skeletal muscle loss during immune checkpoint inhibitor therapy was associated with poorer survival in advanced HCC (13). Because SMI quantifies muscle quantity rather than function, muscle mass and strength may be impaired discordantly (10).

HGS is a simple, low-cost measure of grip strength that may complement CT-derived SMI, because HGS assesses force generation whereas SMI primarily quantifies muscle quantity (10, 11, 14). In cirrhosis, reduced HGS was independently associated with mortality, whereas CT-derived body-composition measures were not retained in adjusted models (15). Low HGS has also been associated with poorer overall survival (OS) in patients with unresectable HCC treated with atezolizumab plus bevacizumab (16). Our previous analysis of a larger institutional cohort showed poorer progression-free survival (PFS) and OS among patients with reduced HGS who received HAIC together with targeted therapy and immunotherapy (17). It remains uncertain whether combining HGS and SMI into concordant and discordant phenotypes provides prognostic information beyond either measure alone (18).

We therefore examined the prognostic relevance of joint HGS–SMI phenotypes in patients with unresectable HCC treated with HAIC plus targeted therapy and immunotherapy. Specifically, we compared survival across concordant and discordant phenotypes, assessed the prognostic associations of HGS and SMI, and evaluated the incremental prognostic value of low HGS, low SMI, and the four-phenotype classification beyond established clinical factors for OS.

## 2 Materials and methods

### 2.1 Study design and participants

This single-center retrospective study was a secondary analysis of the propensity score-matched subset from our previously published cohort (17). The source cohort comprised patients who received HAIC plus targeted therapy and immunotherapy at Tongji Hospital, Tongji Medical College, Huazhong University of Science and Technology, between August 2020 and December 2022. Patients were eligible if they had pathologically confirmed HCC, were assessed as surgically unresectable after multidisciplinary review, received HAIC plus targeted therapy and immunotherapy, and had measurable disease. Surgical unresectability was defined as the inability to perform safe curative-intent resection. All 154 patients in the present analysis met these criteria; none was diagnosed solely by clinical criteria or classified solely as oncologically unresectable. Barcelona Clinic Liver Cancer (BCLC) stage alone was not used to determine resectability, and patient-level reasons for surgical unresectability were not separately coded in the source dataset (17).

Of 314 patients with primary liver malignancies screened, 49 were excluded because of a non-HCC primary malignancy (n = 11), loss to follow-up (n = 12), incomplete clinical or imaging data (n = 12), absence of measurable target lesions (n = 10), or Child–Pugh class C liver function (n = 4), leaving 265 eligible patients. In the previous study, these patients were classified as having low HGS (n = 106) or preserved HGS (n = 159), and 1:1 propensity score matching with a caliper of 0.02 yielded 77 patients in each group (17).

The present analysis used the same 154-patient matched subset; no additional matching was performed. All 154 patients had continuous baseline HGS measurements and evaluable pretreatment CT images at the third lumbar vertebral level for SMI assessment. For the present analysis, HGS status was recalculated directly from the continuous measurements using the prespecified thresholds of < 28 kg in men and < 18 kg in women. Reapplication of these thresholds reclassified one patient relative to the historical binary grouping, yielding 76 patients with low HGS and 78 with preserved HGS. These patients were subsequently classified into the four HGS–SMI phenotypes (**Figure 1**).

**Figure 1:**
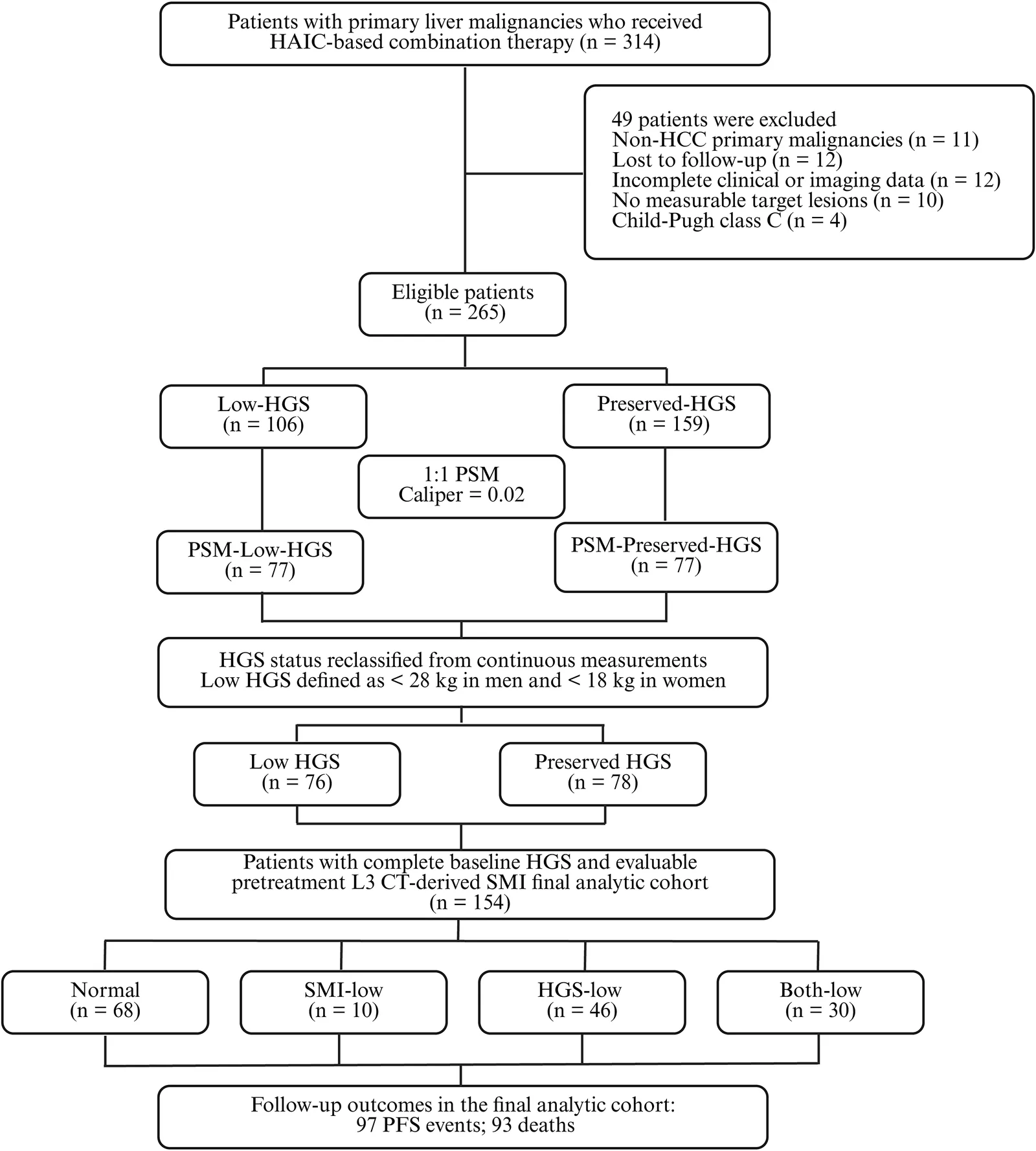
Study cohort and muscle phenotype classification. One record was discordant between the historical binary HGS classification and the continuous HGS measurement; therefore, reclassification using the sex-specific cutoffs of < 28 kg in men and < 18 kg in women yielded 76 patients with low HGS and 78 with preserved HGS. Patients were classified as Normal (normal HGS and SMI), SMI-low (low SMI with preserved HGS), HGS-low (low HGS with normal SMI), or Both-low (low HGS and low SMI). All 154 patients were included in the descriptive, Kaplan–Meier, and multivariable analyses; 97 PFS events and 93 deaths occurred. CT, computed tomography; HAIC, hepatic arterial infusion chemotherapy; HCC, hepatocellular carcinoma; HGS, handgrip strength; L3, third lumbar vertebral level; PFS, progression-free survival; PSM, propensity score matching; SMI, skeletal muscle index.

The study was approved by the Ethics Committee of Tongji Hospital, Tongji Medical College, Huazhong University of Science and Technology (approval no. TJ-IRB202403001), and was conducted in accordance with the Declaration of Helsinki. Because this retrospective study used de-identified routinely collected clinical data, the requirement for written informed consent was waived by the ethics committee.

### 2.2 Treatment and follow-up

Patients received mFOLFOX-based HAIC with an immune checkpoint inhibitor and a targeted agent. Individual targeted agents and immune checkpoint inhibitors were abstracted from the treatment records and are detailed in **Supplementary Table S1**. HAIC was generally repeated every 4–6 weeks until disease progression, unacceptable toxicity, treatment modification, or death. The treating multidisciplinary team determined dose adjustments and subsequent treatment according to tumor characteristics, liver function, response, and tolerability.

Contrast-enhanced CT or magnetic resonance imaging was generally performed at approximately 6-week intervals. Tumor response was assessed according to the modified Response Evaluation Criteria in Solid Tumors (mRECIST) (19). Best overall response was defined as the best mRECIST response recorded from treatment initiation until progression. The objective response rate (ORR) included complete or partial response, whereas the disease control rate (DCR) additionally included stable disease. All 154 patients had at least one post-baseline imaging assessment and were evaluable for response. Follow-up continued until January 24, 2024.

### 2.3 Assessment of HGS, SMI, and muscle phenotypes

Baseline HGS was measured before treatment using a digital hand dynamometer (EH101, Xiangshan Camry Electronic Co., Ltd., China). Participants were seated with the shoulder adducted and neutrally rotated, the elbow flexed at 90°, and the forearm and wrist in a neutral position. Three maximum-effort grip trials were performed using the dominant hand, with a 1-minute rest interval between trials, and the highest value was recorded. A separate familiarization trial and the use of standardized verbal encouragement were not documented in the retrospective records. Low HGS was defined *a priori* as < 28 kg in men and < 18 kg in women, consistent with the second-edition Japan Society of Hepatology criteria for liver disease and the Asian Working Group for Sarcopenia 2019 consensus for Asian populations (20, 21). These thresholds were selected before the present analyses rather than derived from the survival outcomes of the current cohort. The same sex-specific thresholds were used in our previous source-cohort analysis of patients with unresectable HCC receiving HAIC combined with targeted therapy and immunotherapy (17).

Two investigators evaluated abdominal CT images obtained within 2 weeks before treatment. Discrepancies were resolved by consensus after joint review. Skeletal muscle area was measured at the third lumbar vertebral level using ImageJ software (version 1.53; National Institutes of Health, Bethesda, MD, USA). Muscle tissue, including the psoas, paraspinal, and abdominal wall muscles, was identified using an attenuation range of −29 to 150 Hounsfield units (22, 23). SMI was calculated by dividing total L3 skeletal muscle area by height squared: SMI (cm^2^/m^2^) = total L3 skeletal muscle area (cm^2^)/height^2^ (m^2^). Low SMI was defined as < 42 cm^2^/m^2^ in men and < 38 cm^2^/m^2^ in women (20).

Four mutually exclusive phenotypes were defined: Normal, with normal HGS and normal SMI; SMI-low, with low SMI and normal HGS; HGS-low, with low HGS and normal SMI; and Both-low, with both low HGS and low SMI.

### 2.4 Outcomes

The primary outcomes were progression-free survival (PFS) and overall survival (OS). PFS was calculated from treatment initiation to radiological progression or death from any cause, whichever occurred first. Patients without an event were censored at the last evaluable disease assessment.

OS was calculated from treatment initiation to death from any cause; patients who remained alive were censored at the last follow-up. Secondary outcomes were ORR and DCR. The incremental prognostic value of HGS, SMI, and the four-phenotype classification was evaluated as a secondary analysis.

### 2.5 Statistical analysis

Continuous variables were summarized as medians and interquartile ranges (IQRs) and compared using the Kruskal–Wallis test. Categorical variables were summarized as counts and percentages and compared using Fisher’s exact test, with the Fisher–Freeman–Halton extension for r × c contingency tables.

PFS and OS were estimated using the Kaplan–Meier method and compared across phenotypes using the log-rank test. Phenotype-specific 12-month PFS and OS estimates with 95% confidence intervals (CIs) were obtained from the Kaplan–Meier curves. Separate multivariable Cox proportional hazards models were fitted for PFS and OS to estimate adjusted hazard ratios (HRs) and 95% CIs. Normal was the reference phenotype. Models were adjusted for the prespecified covariates of age, sex, Eastern Cooperative Oncology Group performance status (ECOG PS), Child–Pugh class, maximum tumor diameter, tumor number, portal vein tumor thrombosis (PVTT), extrahepatic spread, and previous HCC treatment. These covariates were selected *a priori* to represent demographic characteristics, functional status, hepatic reserve, tumor burden and dissemination, and treatment history while limiting model complexity in relation to the number of observed deaths.

The proportional hazards assumption for each Cox model was evaluated using Schoenfeld residuals. To explicitly accommodate time-varying effects in the OS analysis, an exploratory extended Cox model was fitted using the same adjustment covariates as the primary model. Time-by-covariate interactions with log-transformed follow-up time were included for muscle phenotype, sex, and extrahepatic spread, which showed evidence of nonproportionality in the Schoenfeld residual analysis. Log time was centered at 12 months [log(t/12)], and time-specific adjusted HRs were estimated at 6, 12, and 24 months. Time interactions were evaluated using likelihood-ratio tests, including a joint test with 3 degrees of freedom for muscle phenotype. Restricted mean survival time (RMST) was estimated at 12, 18, and 24 months, and differences versus the Normal phenotype were calculated with 95% CIs obtained from 1,000 stratified bootstrap resamples.

Incremental prognostic value for OS was evaluated by separately adding low SMI, low HGS, or the four muscle phenotypes to the clinical model comprising the prespecified covariates listed above. Harrell’s concordance index (C-index) quantified model discrimination, and nested models were compared using likelihood-ratio tests. The 95% CIs for the C-index and difference in C-index (ΔC-index) were estimated using 500 bootstrap resamples. Internal validation was additionally performed using 2,000 paired bootstrap resamples. In each resample, every model was fitted in the bootstrap sample and its C-index was evaluated in both the bootstrap sample and the original cohort. Mean optimism was calculated as the average difference between bootstrap-sample and original-cohort performance and was subtracted from the apparent C-index. Optimism-corrected ΔC-indices were calculated using paired within-resample differences between each augmented model and its corresponding reference model. As an exploratory analysis, time-dependent discrimination was additionally assessed at 12, 18, and 24 months using inverse probability of censoring weighting (IPCW)-based time-dependent areas under the receiver operating characteristic curve (AUCs). Pairwise differences in AUC (ΔAUC) between models were calculated at each time point, with 95% CIs estimated using 200 bootstrap resamples.

As an additional sensitivity analysis, a parsimonious reference model included ECOG PS, Child–Pugh class, maximum tumor diameter, tumor number, PVTT, extrahepatic spread, and alpha-fetoprotein (AFP) ≥ 400 ng/mL. This model represented functional status, hepatic reserve, tumor burden and dissemination, and an established HCC biomarker using fewer parameters. The same paired bootstrap optimism-correction procedure was applied to this model and its augmented models. In sensitivity analyses, HGS and SMI were standardized and initially modeled separately, with HRs expressed per 1-standard deviation (SD) decrease. To evaluate their independent prognostic contributions, standardized HGS and SMI were subsequently entered simultaneously into the same multivariable Cox models with adjustment for the prespecified clinical covariates. An exploratory HGS-by-SMI interaction term was then added to examine multiplicative interaction, and model fit with and without the interaction term was compared using a likelihood-ratio test. The phenotype analyses were also repeated in the male-only subset and in patients receiving lenvatinib plus an immune checkpoint inhibitor; HAIC exposure was summarized by the recorded number of cycles. All tests were two-sided, with *P* < 0.05 indicating statistical significance. Analyses were performed using R version 4.6.1 (R Foundation for Statistical Computing, Vienna, Austria). All included patients had complete data for the prespecified covariates, and no imputation was performed.

## 3 Results

### 3.1 Baseline and treatment characteristics

All 154 patients from the previously generated propensity score-matched subset were included in the present analysis. Recalculation from the continuous HGS measurements classified 76 patients as having low HGS and 78 as having preserved HGS. Joint classification by HGS and SMI yielded 68 (44.2%) Normal, 10 (6.5%) SMI-low, 46 (29.9%) HGS-low, and 30 (19.5%) Both-low patients (**Figure 1**). All 154 patients had pathologically confirmed HCC and were classified as surgically unresectable; 43 had BCLC stage A or B.

The median age was 54.0 years (IQR, 47.0–61.0), and 134 patients (87.0%) were men. ECOG PS scores were 0, 1, and 2 in 100 (64.9%), 52 (33.8%), and 2 (1.3%) patients, respectively. Child–Pugh class A was present in 126 patients (81.8%), and hepatitis B virus-related HCC in 113 (73.4%). The median maximum tumor diameter was 9.8 cm (IQR, 7.8–12.9). Multiple tumors and PVTT were each present in 96 patients (62.3%), and 31 (20.1%) had extrahepatic spread.

BCLC stage C disease was present in 111 patients (72.1%), and 111 (72.1%) were treatment-naive. The predominant systemic regimen was lenvatinib plus an immune checkpoint inhibitor, used in 127 patients (82.5%); individual systemic regimens are detailed in **Supplementary Table S1**. HAIC cycle counts were available for 153 patients; the median exposure was 2 cycles (IQR, 1–3; range, 1–6). Sex distribution differed across phenotypes (*P* < 0.001), whereas no statistically significant differences were observed in the other baseline or treatment characteristics (all *P* > 0.05; **Table 1**).

**Table 1:** Baseline and treatment characteristics according to muscle phenotype.

Variable	Overall (n = 154)	Normal (n = 68)	SMI-low (n = 10)	HGS-low (n = 46)	Both-low (n = 30)	*P* value
Age, years	54.0 (47.0–61.0)	53.0 (46.8–63.0)	54.0 (42.0–58.8)	53.0 (47.0–61.0)	55.0 (48.2–61.8)	0.894
Sex						< 0.001
Female	20 (13.0%)	6 (8.8%)	5 (50.0%)	9 (19.6%)	0 (0.0%)	
Male	134 (87.0%)	62 (91.2%)	5 (50.0%)	37 (80.4%)	30 (100.0%)	
BMI, kg/m²	21.9 (20.2–24.0)	21.8 (20.6–24.0)	20.4 (19.6–22.6)	22.0 (19.4–24.4)	22.2 (20.6–23.6)	0.569
ECOG PS						0.340
0	100 (64.9%)	46 (67.6%)	5 (50.0%)	28 (60.9%)	21 (70.0%)	
1	52 (33.8%)	22 (32.4%)	4 (40.0%)	17 (37.0%)	9 (30.0%)	
2	2 (1.3%)	0 (0.0%)	1 (10.0%)	1 (2.2%)	0 (0.0%)	
HCC etiology						0.543
HBV	113 (73.4%)	47 (69.1%)	6 (60.0%)	36 (78.3%)	24 (80.0%)	
HCV	3 (1.9%)	1 (1.5%)	0 (0.0%)	1 (2.2%)	1 (3.3%)	
Other/unknown	38 (24.7%)	20 (29.4%)	4 (40.0%)	9 (19.6%)	5 (16.7%)	
Child–Pugh class						0.211
A	126 (81.8%)	58 (85.3%)	6 (60.0%)	36 (78.3%)	26 (86.7%)	
B	28 (18.2%)	10 (14.7%)	4 (40.0%)	10 (21.7%)	4 (13.3%)	
AFP, ng/mL	440.2 (18.9–31251.5)	396.1 (13.7–16760.0)	60500.0 (953.8–60500.0)	270.6 (24.7–22264.8)	911.8 (12.8–23684.8)	0.899
Maximum tumor diameter, cm	9.8 (7.8–12.9)	9.2 (7.2–12.7)	9.4 (7.9–10.6)	9.9 (8.5–14.6)	11.6 (8.1–12.5)	0.268
Tumor number						0.106
Solitary	58 (37.7%)	27 (39.7%)	3 (30.0%)	12 (26.1%)	16 (53.3%)	
Multiple	96 (62.3%)	41 (60.3%)	7 (70.0%)	34 (73.9%)	14 (46.7%)	
PVTT						0.524
Absent	58 (37.7%)	26 (38.2%)	4 (40.0%)	20 (43.5%)	8 (26.7%)	
Present	96 (62.3%)	42 (61.8%)	6 (60.0%)	26 (56.5%)	22 (73.3%)	
Extrahepatic spread						0.977
Absent	123 (79.9%)	54 (79.4%)	8 (80.0%)	36 (78.3%)	25 (83.3%)	
Present	31 (20.1%)	14 (20.6%)	2 (20.0%)	10 (21.7%)	5 (16.7%)	
BCLC stage						0.806
A	16 (10.4%)	7 (10.3%)	2 (20.0%)	5 (10.9%)	2 (6.7%)	
B	27 (17.5%)	10 (14.7%)	2 (20.0%)	10 (21.7%)	5 (16.7%)	
C	111 (72.1%)	51 (75.0%)	6 (60.0%)	31 (67.4%)	23 (76.7%)	
Previous HCC treatment						0.093
No	111 (72.1%)	45 (66.2%)	10 (100.0%)	32 (69.6%)	24 (80.0%)	
Yes	43 (27.9%)	23 (33.8%)	0 (0.0%)	14 (30.4%)	6 (20.0%)	
Systemic treatment regimen						0.898
Lenvatinib + ICI	127 (82.5%)	56 (82.4%)	9 (90.0%)	39 (84.8%)	23 (76.7%)	
Other TKI + ICI	17 (11.0%)	9 (13.2%)	1 (10.0%)	4 (8.7%)	3 (10.0%)	
Anti-VEGF antibody + ICI	3 (1.9%)	1 (1.5%)	0 (0.0%)	1 (2.2%)	1 (3.3%)	
Other/unspecified combinations	7 (4.5%)	2 (2.9%)	0 (0.0%)	2 (4.3%)	3 (10.0%)	
HGS, kg	26.9 (22.2–34.5)	35.0 (30.9–39.0)	27.7 (23.2–28.8)	23.0 (19.0–26.0)	22.4 (20.4–24.9)	—
SMI, cm^2^/m^2^	48.4 (41.0–54.3)	55.1 (52.7–57.9)	36.8 (28.8–38.0)	47.2 (44.0–49.0)	37.0 (35.0–40.1)	—

Data are presented as median (IQR) or n (%). Continuous variables were compared using the Kruskal–Wallis test, and categorical variables were compared using Fisher’s exact test, with the Fisher–Freeman–Halton extension for contingency tables larger than 2 × 2. HGS and SMI were not compared across groups because they defined the phenotypes. AFP values ≥ 60, 500 ng/mL were recorded as 60,500 ng/mL, the upper laboratory reporting limit. AFP, alpha-fetoprotein; BCLC, Barcelona Clinic Liver Cancer; BMI, body mass index; ECOG PS, Eastern Cooperative Oncology Group performance status; HBV, hepatitis B virus; HCC, hepatocellular carcinoma; HCV, hepatitis C virus; HGS, handgrip strength; ICI, immune checkpoint inhibitor; IQR, interquartile range; PVTT, portal vein tumor thrombosis; SMI, skeletal muscle index; TKI, tyrosine kinase inhibitor; VEGF, vascular endothelial growth factor.

### 3.2 Tumor response

All 154 patients were evaluable for best overall response. ORR was 51.5% (35/68) in Normal, 30.0% (3/10) in SMI-low, 39.1% (18/46) in HGS-low, and 50.0% (15/30) in Both-low, with no statistically significant difference across phenotypes (*P* = 0.430). The corresponding DCRs were 92.6%, 90.0%, 95.7%, and 96.7% (*P* = 0.623; **Table 2**).

**Table 2:** Tumor response according to muscle phenotype.

Muscle phenotype	n	ORR, n (%) [95% CI]	DCR, n (%) [95% CI]
Normal	68	35 (51.5) [39.0–63.8]	63 (92.6) [83.7–97.6]
SMI-low	10	3 (30.0) [6.7–65.2]	9 (90.0) [55.5–99.7]
HGS-low	46	18 (39.1) [25.1–54.6]	44 (95.7) [85.2–99.5]
Both-low	30	15 (50.0) [31.3–68.7]	29 (96.7) [82.8–99.9]
Overall *P* value		0.430	0.623

Values are n (%) with exact binomial 95% CIs. Overall differences among phenotypes were assessed using Fisher’s exact test. ORR and DCR were based on best overall response according to mRECIST, and all 154 patients were evaluable. CI, confidence interval; DCR, disease control rate; HGS, handgrip strength; mRECIST, modified Response evaluation criteria in solid tumors; ORR, objective response rate; SMI, skeletal muscle index.

### 3.3 Survival outcomes

Kaplan–Meier curves for both PFS and OS differed significantly across the four phenotypes (both log-rank *P* < 0.001; **Figure 2**). The estimated 12-month PFS rates were 62.2% (95% CI, 49.3–72.7) for Normal, 90.0% (47.3–98.5) for SMI-low, 23.9% (12.9–36.9) for HGS-low, and 39.0% (21.6–56.0) for Both-low. The corresponding 12-month OS rates were 82.2% (70.8–89.5), 90.0% (47.3–98.5), 37.0% (23.4–50.6), and 39.7% (22.5–56.5), respectively. The median follow-up duration estimated by reverse Kaplan-Meier analysis was 22.1 months. During follow-up, 97 PFS events and 93 deaths occurred. By phenotype, the numbers of PFS events and deaths were 34 and 31 in Normal, 6 and 6 in SMI-low, 38 and 37 in HGS-low, and 19 and 19 in Both-low, respectively.

**Figure 2:**
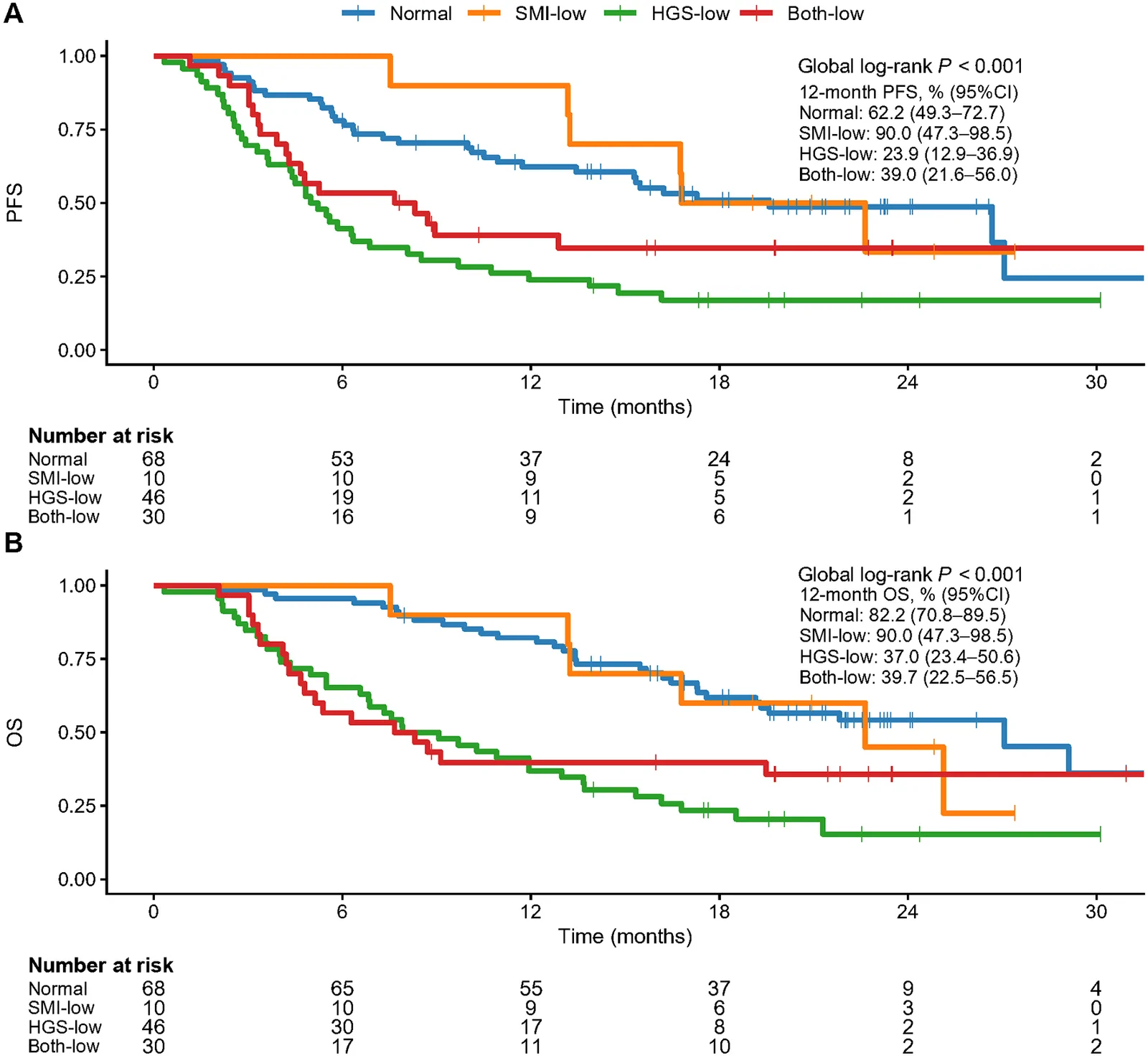
Kaplan–Meier estimates of progression-free survival and overall survival according to muscle phenotypes. **(A)** Progression-free survival and **(B)** overall survival according to muscle phenotype. Tick marks indicate censored observations, and numbers at risk are shown below each panel. Global differences were assessed using the log-rank test. Phenotype-specific 12-month PFS and OS estimates are presented as percentages with 95% CIs in the corresponding panels. CI, confidence interval; HGS, handgrip strength; OS, overall survival; PFS, progression-free survival; SMI, skeletal muscle index.

After multivariable adjustment, HGS-low was associated with shorter PFS than Normal (HR, 2.81; 95% CI, 1.71–4.60; *P* < 0.001). Both-low was also associated with shorter PFS (HR, 2.53; 95% CI, 1.40–4.56; *P* = 0.002), whereas SMI-low was not significantly associated with PFS (HR, 0.79; 95% CI, 0.29–2.16; *P* = 0.644). Larger maximum tumor diameter (HR per 1-cm increase, 1.05; 95% CI, 1.00–1.11; *P* = 0.037), multiple tumors (HR, 2.63; 95% CI, 1.58–4.39; *P* < 0.001), and previous HCC treatment (HR, 1.89; 95% CI, 1.17–3.06; *P* = 0.010) were also associated with shorter PFS. Age, sex, ECOG PS, Child–Pugh class, PVTT, and extrahepatic spread were not significantly associated with PFS (**Table 3**; **Figure 3A**).

**Table 3:** Multivariable Cox regression analysis for progression-free survival.

Variable	Adjusted HR	95% CI	*P* value
Muscle phenotype			
Normal	1.00	Reference	—
SMI-low	0.79	0.29–2.16	0.644
HGS-low	2.81	1.71–4.60	< 0.001
Both-low	2.53	1.40–4.56	0.002
Sex, female vs male	0.91	0.45–1.85	0.797
Age, per 1-year increase	1.01	0.99–1.02	0.494
ECOG PS, per 1-point increase	1.45	0.95–2.20	0.084
Child–Pugh class, B vs A	1.37	0.79–2.38	0.255
Maximum tumor diameter, per 1-cm increase	1.05	1.00–1.11	0.037
Tumor number, multiple vs solitary	2.63	1.58–4.39	< 0.001
PVTT, present vs absent	1.11	0.72–1.71	0.633
Extrahepatic spread, present vs absent	1.33	0.77–2.30	0.308
Previous HCC treatment, yes vs no	1.89	1.17–3.06	0.010

Adjusted HRs were estimated using a multivariable Cox proportional hazards model including 154 patients and 97 PFS events. Normal was the reference phenotype. Normal indicates normal HGS and SMI; SMI-low, low SMI with normal HGS; HGS-low, low HGS with normal SMI; and Both-low, low HGS and low SMI. The proportional hazards assumption was assessed using Schoenfeld residuals, with the results reported in **Supplementary Table S2**. The muscle phenotype effect showed evidence of time variation in the OS model; therefore, the OS HRs in **Table 4** should be interpreted as overall associations across follow-up rather than constant effects at every time point. CI, confidence interval; ECOG PS, Eastern cooperative oncology group performance status; HCC, hepatocellular carcinoma; HGS, handgrip strength; HR, hazard ratio; OS, overall survival; PFS, progression-free survival; PVTT, portal vein tumor thrombosis; SMI, skeletal muscle index.

**Figure 3:**
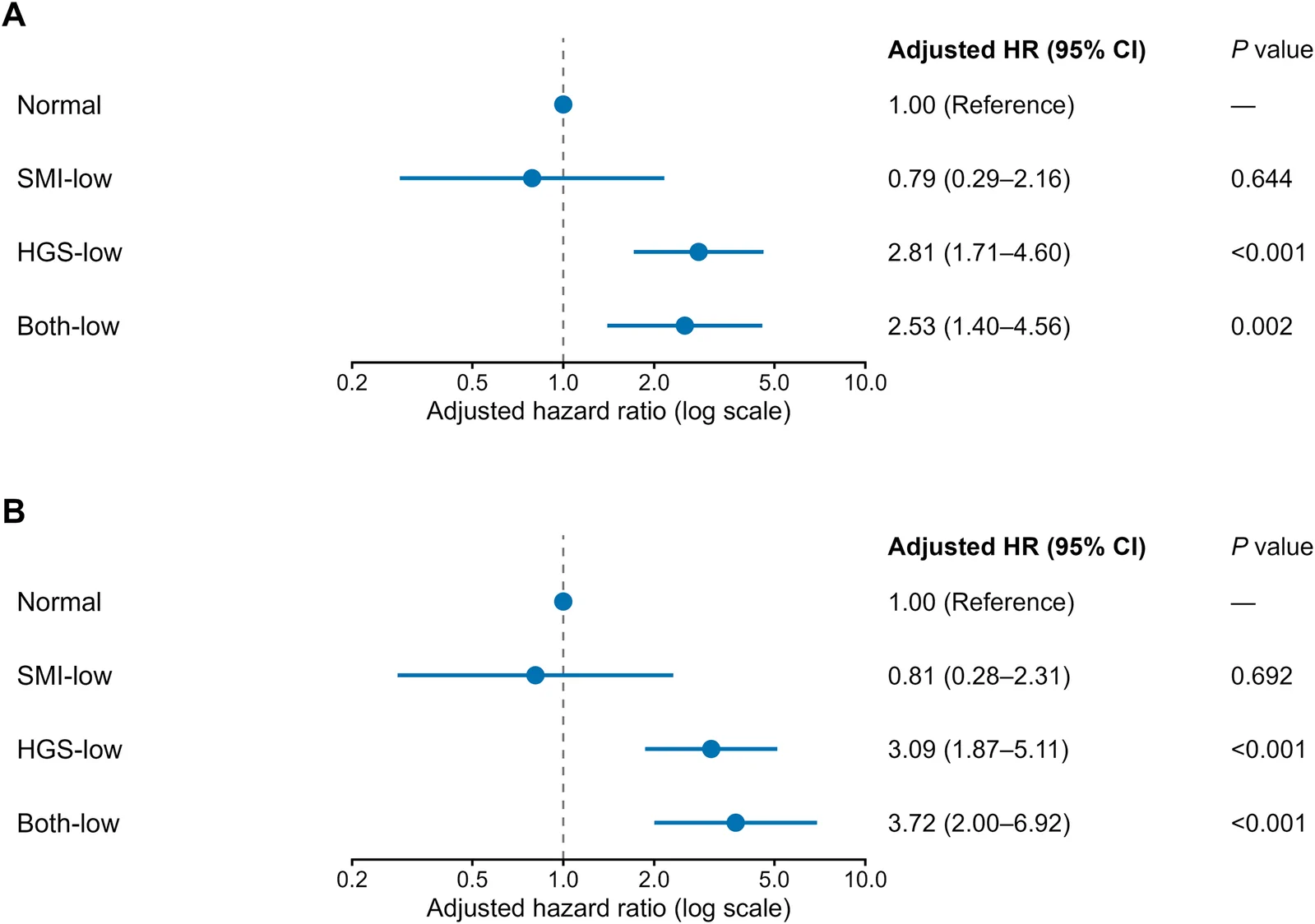
Adjusted associations of muscle phenotypes with progression-free survival and overall survival. Forest plots show adjusted HRs and 95% CIs for **(A)** PFS and **(B)** OS, with the Normal phenotype as the reference. Models included 154 patients and were adjusted for age, sex, ECOG PS, Child–Pugh class, maximum tumor diameter, tumor number, PVTT, extrahepatic spread, and previous HCC treatment. Because the muscle phenotype effect varied over time in the OS model, the HRs displayed in panel **(B)** summarize overall associations across follow-up; time-specific estimates from the extended Cox model are reported in **Supplementary Table S3**. CI, confidence interval; ECOG PS, Eastern cooperative oncology group performance status; HCC, hepatocellular carcinoma; HGS, handgrip strength; HR, hazard ratio; OS, overall survival; PFS, progression-free survival; PVTT, portal vein tumor thrombosis; SMI, skeletal muscle index.

In the multivariable OS model, HGS-low (HR, 3.09; 95% CI, 1.87–5.11; *P* < 0.001) and Both-low (HR, 3.72; 95% CI, 2.00–6.92; *P* < 0.001) were associated with shorter OS than Normal, whereas SMI-low was not significantly associated with OS (HR, 0.81; 95% CI, 0.28–2.31; *P* = 0.692). Higher ECOG PS (HR per 1-point increase, 1.55; 95% CI, 1.01–2.38; *P* = 0.043), Child–Pugh class B versus A (HR, 2.01; 95% CI, 1.16–3.48; *P* = 0.012), and multiple tumors (HR, 2.35; 95% CI, 1.39–3.99; *P* = 0.002) were also associated with shorter OS. Maximum tumor diameter was not significantly associated with OS (HR per 1-cm increase, 1.05; 95% CI, 1.00–1.10; *P* = 0.053). Age, sex, PVTT, extrahepatic spread, and previous HCC treatment were also not significantly associated with OS (**Table 4**; **Figure 3B**).

**Table 4:** Multivariable Cox regression analysis for overall survival.

Variable	Adjusted HR	95% CI	*P* value
Muscle phenotype			
Normal	1.00	Reference	—
SMI-low	0.81	0.28–2.31	0.692
HGS-low	3.09	1.87–5.11	< 0.001
Both-low	3.72	2.00–6.92	< 0.001
Sex, female vs male	0.95	0.47–1.92	0.893
Age, per 1-year increase	1.016	0.998–1.034	0.088
ECOG PS, per 1-point increase	1.55	1.01–2.38	0.043
Child–Pugh class, B vs A	2.01	1.16–3.48	0.012
Maximum tumor diameter, per 1-cm increase	1.05	1.00–1.10	0.053
Tumor number, multiple vs solitary	2.35	1.39–3.99	0.002
PVTT, present vs absent	0.97	0.63–1.51	0.897
Extrahepatic spread, present vs absent	1.48	0.86–2.53	0.153
Previous HCC treatment, yes vs no	1.53	0.93–2.53	0.096

Adjusted HRs were estimated using a multivariable Cox proportional hazards model including 154 patients and 93 deaths. Normal was the reference phenotype. Normal indicates normal HGS and SMI; SMI-low, low SMI with normal HGS; HGS-low, low HGS with normal SMI; and Both-low, low HGS and low SMI. The proportional hazards assumption was assessed using Schoenfeld residuals, with the results reported in **Supplementary Table S2**. Because the muscle phenotype effect showed evidence of time variation in the OS model, HRs should be interpreted as average associations over the observed follow-up rather than as constant effects at every time point. Time-specific HRs from the exploratory extended Cox model are reported in **Supplementary Table S3**. CI, confidence interval; ECOG PS, Eastern cooperative oncology group performance status; HCC, hepatocellular carcinoma; HGS, handgrip strength; HR, hazard ratio; OS, overall survival; PVTT, portal vein tumor thrombosis; SMI, skeletal muscle index.

For PFS, the global Schoenfeld residual test was not significant (*P* = 0.234). The phenotype term did not show significant evidence of nonproportionality (*P* = 0.051), although sex did (*P* = 0.011). For OS, the global test was also not significant (*P* = 0.107), whereas muscle phenotype (*P* = 0.014), sex (*P* = 0.004), and extrahepatic spread (*P* = 0.036) showed evidence of nonproportionality (**Supplementary Table S2**). This finding indicates that the relative hazards associated with muscle phenotype may vary over time; therefore, the Cox HRs for OS should be interpreted as overall associations averaged across the observed follow-up rather than as constant effects at every time point. An exploratory extended Cox model was fitted with interactions between log-transformed follow-up time and muscle phenotype, sex, and extrahepatic spread. The overall phenotype-by-time interaction was significant (*P* = 0.042), as was the sex-by-time interaction (*P* = 0.036), whereas the extrahepatic-spread-by-time interaction was not significant under the specified log-time function (*P* = 0.430). Compared with the Normal phenotype, the adjusted HRs for OS associated with HGS-low were 4.82 (95% CI, 2.47–9.40), 2.44 (95% CI, 1.31–4.53), and 1.24 (95% CI, 0.42–3.62) at 6, 12, and 24 months, respectively. The corresponding HRs for Both-low were 5.58 (95% CI, 2.56–12.17), 2.40 (95% CI, 1.06–5.45), and 1.03 (95% CI, 0.28–3.78). These estimates indicate that the excess mortality hazards associated with the low-HGS phenotypes were greatest during earlier follow-up and attenuated over time (**Supplementary Table S3**).

### 3.4 Incremental prognostic value

For OS, the clinical model had a C-index of 0.658 (95% CI, 0.625–0.735). Adding low SMI yielded a C-index of 0.669 (95% CI, 0.637–0.745) and a ΔC-index of 0.011 (95% CI, −0.005 to 0.039), but did not significantly improve model fit (likelihood-ratio *P* = 0.186).

The C-index increased to 0.731 (95% CI, 0.692–0.792) after adding low HGS, with a ΔC-index of 0.074 (95% CI, 0.027–0.117). Model fit also improved significantly (likelihood-ratio *P* < 0.001). Adding the four phenotypes yielded a C-index of 0.733 (95% CI, 0.699–0.795) and a ΔC-index of 0.075 (95% CI, 0.028–0.119), with a significant improvement in model fit (likelihood-ratio *P* < 0.001). However, the four-phenotype model did not further improve model fit beyond the clinical model plus low HGS in the direct nested comparison (likelihood-ratio *P* = 0.798; **Table 5**).

**Table 5:** Incremental prognostic value of muscle measures for overall survival beyond the clinical model.

Model	Apparent C-index	Optimism-corrected C-index	Apparent ΔC-index	Optimism-corrected ΔC-index	Likelihood-ratio *P* value
Clinical model	0.658	0.625	Reference	Reference	—
Clinical model + low SMI	0.669	0.632	0.011	0.007	0.186
Clinical model + low HGS	0.731	0.705	0.074	0.080	< 0.001
Clinical model + four-phenotype classification	0.733	0.701	0.075	0.076	< 0.001
Direct comparison: clinical model + four-phenotype classification vs clinical model + low HGS	—	—	—	−0.005	0.798

The clinical model included age, sex, ECOG PS, Child–Pugh class, maximum tumor diameter, tumor number, PVTT, extrahepatic spread, and previous HCC treatment. Optimism-corrected estimates were obtained using 2,000 paired bootstrap resamples. The direct-comparison row compares the four-phenotype model with the low-HGS model. C-index, concordance index; ΔC-index, difference in concordance index; ECOG PS, Eastern cooperative oncology group performance status; HCC, hepatocellular carcinoma; HGS, handgrip strength; PVTT, portal vein tumor thrombosis; SMI, skeletal muscle index.

After internal validation, the optimism-corrected C-index was 0.625 for the clinical model, 0.632 after adding low SMI, 0.705 after adding low HGS, and 0.701 after adding the four-phenotype classification. The corresponding optimism-corrected ΔC-indices versus the clinical model were 0.007, 0.080, and 0.076, respectively. The optimism-corrected ΔC-index for the direct comparison of the four-phenotype model with the low-HGS model was −0.005, consistent with the absence of additional improvement beyond low HGS alone (**Table 5**).

In exploratory time-dependent discrimination analyses, ΔAUCs between the four-phenotype model and the clinical model plus low HGS were 0.002, 0.003, and 0.009 at 12, 18, and 24 months, respectively. Compared with the clinical model plus low SMI, the corresponding ΔAUC values were 0.116, 0.066, and 0.034 (**Supplementary Table S4**).

### 3.5 Complementary and sensitivity analyses

Because the muscle phenotype effect in the OS model showed evidence of time variation, RMST estimates were emphasized as complementary measures that do not require the proportional hazards assumption. At the 24-month horizon, RMST for PFS was 7.57 months shorter in HGS-low than in Normal (95% CI, −10.55 to −4.33) and 4.39 months shorter in Both-low (95% CI, −8.25 to −0.47). In SMI-low, RMST for PFS was 2.43 months longer than in Normal (95% CI, −1.98 to 6.41). RMST for OS was 7.92 months shorter in HGS-low (95% CI, −10.47 to −5.32) and 6.50 months shorter in Both-low (95% CI, −10.28 to −2.82) than in Normal. In SMI-low, RMST for OS was 0.36 months longer than in Normal (95% CI, −3.59 to 3.99; **Supplementary Tables S5**, **S6**).

In continuous-variable sensitivity analyses, a 1-SD decrease in HGS was associated with shorter PFS (HR, 2.00; 95% CI, 1.52–2.62; *P* < 0.001) and OS (HR, 2.48; 95% CI, 1.85–3.32; *P* < 0.001). A 1-SD decrease in SMI was not significantly associated with PFS (HR, 1.13; 95% CI, 0.92–1.39; *P* = 0.241) but was associated with shorter OS (HR, 1.29; 95% CI, 1.05–1.58; *P* = 0.016; **Supplementary Table S7**). HGS and SMI were correlated (Pearson r = 0.710). When standardized HGS and SMI were entered simultaneously into the same multivariable models, a 1-SD decrease in HGS remained associated with shorter PFS (HR, 2.53; 95% CI, 1.81–3.55; *P* < 0.001) and OS (HR, 3.05; 95% CI, 2.11–4.41; *P* < 0.001). In the same models, the HRs per 1-SD decrease in SMI were 0.70 (95% CI, 0.52–0.95; *P* = 0.024) for PFS and 0.75 (95% CI, 0.54–1.03; *P* = 0.071) for OS. There was no evidence of an HGS-by-SMI interaction for PFS (interaction HR, 1.07; 95% CI, 0.80–1.42; likelihood-ratio *P* = 0.657) or OS (interaction HR, 1.04; 95% CI, 0.77–1.42; likelihood-ratio *P* = 0.786; **Supplementary Table S8**).

The exploratory male-only analysis included 134 men, with 82 PFS events and 78 deaths. HGS-low was associated with shorter PFS (HR, 3.00; 95% CI, 1.76–5.11; *P* < 0.001) and OS (HR, 2.99; 95% CI, 1.74–5.16; *P* < 0.001). Both-low was also associated with shorter PFS (HR, 2.70; 95% CI, 1.47–4.98; *P* = 0.001) and OS (HR, 3.82; 95% CI, 2.01–7.25; *P* < 0.001). SMI-low was not significantly associated with PFS (HR, 0.66; 95% CI, 0.15–2.89; *P* = 0.579) or OS (HR, 0.74; 95% CI, 0.17–3.30; *P* = 0.698; **Supplementary Table S9**).

AFP was available for all 154 patients; 85 had AFP ≥ 400 ng/mL and 69 had AFP < 400 ng/mL. In the AFP-inclusive parsimonious sensitivity analysis, the optimism-corrected C-index was 0.630 for the reference model, 0.632 after adding low SMI, 0.701 after adding low HGS, and 0.697 after adding the four-phenotype classification. The corresponding optimism-corrected ΔC-indices were 0.002, 0.071, and 0.067, respectively. The four-phenotype model again did not improve model fit beyond low HGS alone (likelihood-ratio *P* = 0.639; optimism-corrected ΔC-index = −0.004; **Supplementary Table S10**).

In the 127 patients receiving lenvatinib plus an immune checkpoint inhibitor (76 PFS events and 72 deaths), the adjusted phenotype associations were consistent with the primary analysis: HGS-low and Both-low were associated with shorter PFS and OS, whereas SMI-low was not (**Supplementary Table S11**).

## 4 Discussion

### 4.1 Principal findings

In this cohort of patients with unresectable HCC treated with HAIC-based combination therapy, low HGS, whether isolated or accompanied by low SMI, remained associated with shorter PFS and OS after multivariable adjustment. The extended Cox analysis further indicated that the excess mortality hazards associated with HGS-low and Both-low were greatest during earlier follow-up and attenuated over time. The 10-patient SMI-low estimates were imprecise for PFS (HR, 0.79; 95% CI, 0.29–2.16) and OS (HR, 0.81; 95% CI, 0.28–2.31). The association with HGS was also observed in the continuous-variable, RMST, and male-only analyses. In models considering the muscle measures separately, continuous SMI was associated with OS but not PFS. In the mutually adjusted continuous models, lower HGS remained strongly associated with both outcomes, whereas the SMI coefficient changed direction, indicating that the independent conditional contributions of HGS and SMI were not equivalent.

Adding low HGS improved discrimination beyond the clinical model, whereas adding low SMI yielded only a small increase in discrimination and did not significantly improve model fit. The four-phenotype classification provided little additional discrimination and did not improve model fit beyond low HGS, suggesting that the incremental prognostic information provided by the four-phenotype classification was largely captured by low HGS. This finding remained consistent after bootstrap optimism correction and in the AFP-inclusive parsimonious sensitivity analysis. These findings should not be interpreted as evidence that muscle mass lacks prognostic relevance. The SMI-low subgroup was small, and lower continuous SMI remained associated with shorter OS.

### 4.2 Comparison with previous studies

Previous studies have associated CT-defined low muscle mass or progressive muscle depletion with adverse survival outcomes in HCC across surgical, locoregional, and systemic treatment settings (13, 24, 25). Preoperative sarcopenia has also been associated with reduced postoperative survival and health-related quality of life after hepatectomy for HCC (26). During liver-directed therapy, early or rapid decreases in SMI during HAIC and other transcatheter intra-arterial treatments have been associated with poorer survival outcomes (27, 28). In HCC patients undergoing transarterial chemoembolization, in-hospital exercise was associated with a more favorable longitudinal change in SMI (29). Separately, Nagamatsu et al. reported that slow walking speed overlapped with low HGS in patients with chronic liver disease and HCC, underscoring that muscle strength and physical function are related but distinct domains (30). In systemic treatment settings, low muscle volume was associated with worse OS during lenvatinib therapy (31), while CT-defined sarcopenia was associated with shorter PFS in patients treated with a programmed cell death protein 1 inhibitor (32). However, definitions, cutoff values, timing of muscle assessment, and treatment populations have varied, and these studies have predominantly focused on muscle quantity rather than direct measures of muscle function. Evidence jointly evaluating HGS and CT-derived muscle mass in patients receiving HAIC with targeted therapy and immunotherapy remains limited.

Our previous study evaluated low HGS in the full 265-patient source cohort and its 154-patient propensity score-matched subset (17). The present secondary analysis used the same 154 matched patients but addressed a distinct question by integrating HGS with CT-derived SMI, defining concordant and discordant muscle phenotypes, and evaluating their incremental prognostic value beyond established clinical factors and low HGS alone. Thus, the present study assessed whether HGS and SMI provided complementary prognostic information rather than reassessing the association between low HGS and survival alone. HGS-low was associated with shorter PFS and OS even when SMI was preserved, whereas estimates for the 10-patient SMI-low subgroup were imprecise.

This pattern is broadly consistent with major sarcopenia frameworks, which recognize muscle strength as a key component of sarcopenia assessment (10, 21). Studies in cirrhosis have similarly suggested that HGS may provide prognostic information more consistently than imaging-based measures of muscle mass (14, 15). In our cohort, a 1-SD decrease in continuous SMI was associated with shorter OS (HR, 1.29; 95% CI, 1.05–1.58; *P* = 0.016), indicating that muscle mass may retain prognostic information. By contrast, the categorical SMI-low phenotype had an imprecise OS estimate (HR, 0.81; 95% CI, 0.28–2.31). These analyses are not directly equivalent: the continuous analysis retained the full range of SMI values across all 154 patients, whereas the SMI-low phenotype comprised only 10 patients with low SMI but preserved HGS, with patients who had both low SMI and low HGS classified separately as Both-low. Dichotomization may have resulted in information loss, particularly for values close to the cut-off, while the small SMI-low subgroup limited statistical power and precision. Therefore, the nonsignificant categorical result should not be interpreted as evidence that low muscle mass lacks prognostic relevance.

HGS and SMI were correlated, and the present cohort was derived from a subset previously matched according to HGS status. Consequently, the inverse conditional coefficient for SMI in the mutually adjusted model may reflect correlation, statistical suppression, and selection related to the matched cohort and should not be interpreted as evidence that lower muscle mass is protective. No multiplicative interaction between standardized HGS and SMI was detected, although the modest sample size limited the power to identify interaction effects.

### 4.3 Potential explanations

Muscle strength and muscle mass represent related but distinct dimensions of muscle health. SMI primarily reflects muscle quantity, whereas HGS reflects muscle function and may be influenced by nutritional, neuromuscular, and systemic factors. In advanced liver disease, systemic inflammation, reduced nutritional intake, hyperammonemia, and physical inactivity can contribute to skeletal muscle dysfunction and wasting (33–35), which may partly explain the stronger and more consistent survival associations observed with HGS than with SMI. Although ORR and DCR did not differ significantly across phenotypes, treatment response may still have contributed to the survival differences; low HGS may additionally reflect host-related vulnerability and limited physiological reserve. Treatment tolerance, cumulative drug doses, complications, and subsequent therapy were not assessed and may also have contributed to the observed associations.

### 4.4 Clinical implications

HGS is a simple, low-cost, noninvasive, and readily repeatable measure that may complement routine risk assessment before HAIC-based combination therapy. Low HGS may identify patients who warrant closer evaluation of nutritional status, physical function, hepatic reserve, and treatment tolerance, but should be interpreted alongside established clinical and tumor-related factors rather than used alone to guide treatment selection. Given the limited incremental prognostic value of the four-phenotype classification beyond low HGS, HGS may serve as a practical initial measure of muscle status, while CT-derived SMI can provide complementary information when abdominal imaging is available. These findings support the prospective evaluation of individualized, supervised exercise and nutritional programs for patients with low HGS, including their effects on treatment tolerance and clinical outcomes.

### 4.5 Strengths and limitations

This study jointly evaluated HGS and CT-derived SMI, allowing concordant and discordant muscle phenotypes to be distinguished. Categorical and continuous analyses, RMST estimates, incremental model comparisons, and a male-only sensitivity analysis provided complementary assessments of the study findings. The cohort also included a clinically relevant population with unresectable HCC receiving HAIC combined with targeted therapy and immunotherapy.

The study has several limitations. Its retrospective, single-center design and modest sample size may limit generalizability, particularly because the SMI-low subgroup included only 10 patients and sex was markedly imbalanced across phenotypes. Because the present analysis was restricted to a previously propensity score-matched subset defined by HGS status, the phenotype distribution and effect estimates may not fully represent the entire source cohort, further limiting generalizability. HGS and SMI were assessed only at baseline, and longitudinal muscle changes, treatment tolerance, and subsequent therapy were not evaluated. Although L3 CT-derived SMI is an established measure of muscle quantity, it does not directly quantify lower-limb muscle mass or assess muscle power, gait-related function, or chair-rise performance. Similarly, HGS reflects grip rather than global muscle strength and was measured only in the dominant hand, which is not necessarily the stronger hand; bilateral measurements were unavailable. Although three trials separated by 1-minute rest intervals were performed, formal familiarization and standardized encouragement were not documented, and potential influences including pain, neuropathy, arthritis, edema, cognition, and motivation were not systematically assessed. Objective physical-performance measures, such as gait speed and chair-rise testing, were not available in this retrospective cohort. Treatment regimens were heterogeneous, and dose reductions, treatment delays, and reasons for discontinuation were not systematically recorded. Although the sex-specific HGS thresholds were based on established Asian and liver-disease criteria and were also used in our previous HCC cohort (17), they have not been independently validated specifically in patients receiving HAIC-based combination therapy. Classification of patients with HGS values close to these thresholds should therefore be interpreted cautiously. In addition, the OS phenotype effect showed evidence of time variation. The extended Cox analysis suggested that the excess mortality hazards associated with the low-HGS phenotypes were greatest during earlier follow-up and subsequently attenuated. The primary Cox HRs should therefore be interpreted as overall associations across follow-up rather than as constant effects at every time point. Estimates at later time points were less precise because fewer patients remained at risk. RMST estimates at prespecified time horizons provided complementary absolute measures that do not depend on the proportional hazards assumption. Finally, the findings require external validation in larger prospective multicenter cohorts.

## 5 Conclusions

Low HGS, with or without low SMI, was associated with poorer survival in patients with unresectable HCC receiving HAIC-based combination therapy. HGS may therefore provide a simple and practical measure for baseline prognostic assessment.

## Data Availability

The raw data supporting the conclusions of this article will be made available by the authors, without undue reservation.
