# Supplementary material for: Muscle strength and muscle mass phenotypes and survival in unresectable hepatocellular carcinoma receiving hepatic arterial infusion chemotherapy-based combination therapy
Source: Front Oncol. 2026 Oct 21;16:1974237. doi: 10.3389/fonc.2026.1974237 (PMC13730874; doi:10.3389/fonc.2026.1974237)
Supplement: Supplementary file 1 [file Table1.docx]

Table S1. Individual systemic treatment regimens documented in the 154-patient cohort

| Targeted or antiangiogenic agent | Immune checkpoint inhibitor | Patients, n |
| --- | --- | --- |
| Lenvatinib | Tislelizumab | 57 |
| Lenvatinib | Camrelizumab | 29 |
| Lenvatinib | Sintilimab | 28 |
| Lenvatinib | Pembrolizumab | 10 |
| Apatinib | Camrelizumab | 8 |
| Donafenib | Tislelizumab | 2 |
| Regorafenib | Pembrolizumab | 2 |
| Apatinib | Sintilimab | 1 |
| Bevacizumab | Atezolizumab | 1 |
| Bevacizumab | Sintilimab | 1 |
| Bevacizumab | Toripalimab | 1 |
| Donafenib | Camrelizumab | 1 |
| Donafenib, subsequently switched to lenvatinib | Sintilimab | 1 |
| Lenvatinib | Durvalumab | 1 |
| Lenvatinib | Toripalimab | 1 |
| Lenvatinib | Unspecified PD-1 inhibitor | 1 |
| Regorafenib | Sintilimab | 1 |
| Regorafenib | Tislelizumab | 1 |
| Regorafenib | Not recorded | 1 |
| Sorafenib | Not recorded | 1 |
| Not recorded | Atezolizumab | 1 |
| Unspecified targeted agent | Camrelizumab | 1 |
| Unspecified targeted agent | Unspecified ICI | 1 |
| Insufficient information in the source record | Insufficient information in the source record | 2 |
| Total |  | 154 |

Agent names were standardized from the source treatment records. “Not recorded” indicates that no concomitant agent was documented, whereas “insufficient information” indicates that the recorded regimen could not be reliably resolved to individual agents. Unavailable drug identities were not imputed. For the broad systemic treatment categories reported in Table 1, patients who switched targeted agents were classified according to the initial recorded targeted agent. ICI, immune checkpoint inhibitor; PD-1, programmed cell death protein 1.

Table S2. Assessment of the proportional hazards assumption using Schoenfeld residuals

| Outcome | Model term | χ² | df | *P* value |
| --- | --- | --- | --- | --- |
| PFS | Muscle phenotype | 7.760 | 3 | 0.051 |
| PFS | Age | 1.515 | 1 | 0.218 |
| PFS | Sex | 6.496 | 1 | 0.011 |
| PFS | ECOG PS | 3.807 | 1 | 0.051 |
| PFS | Child–Pugh class | 3.427 | 1 | 0.064 |
| PFS | Maximum tumor diameter | 0.556 | 1 | 0.456 |
| PFS | Tumor number | 0.040 | 1 | 0.841 |
| PFS | PVTT | 0.359 | 1 | 0.549 |
| PFS | Extrahepatic spread | 1.676 | 1 | 0.195 |
| PFS | Previous HCC treatment | 0.404 | 1 | 0.525 |
| PFS | Global test | 15.138 | 12 | 0.234 |
| OS | Muscle phenotype | 10.626 | 3 | 0.014 |
| OS | Age | 0.897 | 1 | 0.344 |
| OS | Sex | 8.475 | 1 | 0.004 |
| OS | ECOG PS | 2.827 | 1 | 0.093 |
| OS | Child–Pugh class | 0.282 | 1 | 0.595 |
| OS | Maximum tumor diameter | 2.213 | 1 | 0.137 |
| OS | Tumor number | 0.781 | 1 | 0.377 |
| OS | PVTT | 0.574 | 1 | 0.449 |
| OS | Extrahepatic spread | 4.384 | 1 | 0.036 |
| OS | Previous HCC treatment | 2.327 | 1 | 0.127 |
| OS | Global test | 18.302 | 12 | 0.107 |

The proportional hazards assumption was assessed using Schoenfeld residuals for the PFS model (154 patients, 97 events) and OS model (154 patients, 93 deaths). df, degrees of freedom; ECOG PS, Eastern Cooperative Oncology Group performance status; HCC, hepatocellular carcinoma; OS, overall survival; PFS, progression-free survival; PVTT, portal vein tumor thrombosis.

Table S3. Exploratory extended Cox model allowing time-varying effects for overall survival

| Variable | Comparison | Adjusted HR at 6 months (95% CI) | Adjusted HR at 12 months (95% CI) | Adjusted HR at 24 months (95% CI) | *P* value |
| --- | --- | --- | --- | --- | --- |
| Muscle phenotype | SMI-low vs Normal | 0.48 (0.06–3.99) | 0.65 (0.20–2.09) | 0.89 (0.16–4.81) | 0.042* |
|  | HGS-low vs Normal | 4.82 (2.47–9.40) | 2.44 (1.31–4.53) | 1.24 (0.42–3.62) |  |
|  | Both-low vs Normal | 5.58 (2.56–12.17) | 2.40 (1.06–5.45) | 1.03 (0.28–3.78) |  |
| Sex | Female vs male | 0.53 (0.19–1.43) | 1.20 (0.56–2.59) | 2.72 (0.78–9.54) | 0.036 |
| Extrahepatic spread | Yes vs no | 1.33 (0.76–2.31) | 1.60 (0.85–3.01) | 1.93 (0.73–5.13) | 0.430 |

*The extended Cox model included the same adjustment covariates as the primary OS model and additional interactions of muscle phenotype, sex, and extrahepatic spread with log(t/12), where t denotes follow-up time in months. HRs are time-specific estimates at 6, 12, and 24 months. Interaction P values were obtained using likelihood-ratio tests. The muscle phenotype-by-time interaction was evaluated jointly using 3 degrees of freedom. The nonsignificant extrahepatic-spread-by-time interaction under the specified log-time function does not exclude other forms of time variation suggested by the Schoenfeld residual analysis. CI, confidence interval; HGS, handgrip strength; HR, hazard ratio; OS, overall survival; SMI, skeletal muscle index.

Table S4. Exploratory differences in IPCW time-dependent AUC for overall survival

| Time, months | Model comparison | ΔAUC | 95% CI |
| --- | --- | --- | --- |
| 12 | Clinical model + four-phenotype classification vs clinical model + low HGS | 0.002 | −0.009–0.021 |
| 12 | Clinical model + four-phenotype classification vs clinical model + low SMI | 0.116 | 0.026–0.167 |
| 18 | Clinical model + four-phenotype classification vs clinical model + low HGS | 0.003 | −0.009–0.024 |
| 18 | Clinical model + four-phenotype classification vs clinical model + low SMI | 0.066 | 0.006–0.114 |
| 24 | Clinical model + four-phenotype classification vs clinical model + low HGS | 0.009 | −0.020–0.054 |
| 24 | Clinical model + four-phenotype classification vs clinical model + low SMI | 0.034 | −0.047–0.155 |

Model-based risk scores were derived from Cox models adjusted for the clinical covariates listed in Table 5. ΔAUC was calculated as the AUC of the first model minus that of the second model; positive values indicate better discrimination for the first model. The 95% CIs for ΔAUC were estimated using 200 bootstrap resamples. AUC, area under the curve; CI, confidence interval; HGS, handgrip strength; IPCW, inverse probability of censoring weighting; SMI, skeletal muscle index.

Table S5. Restricted mean progression-free survival and differences versus the Normal phenotype at 12, 18, and 24 months

| Time horizon, months | Muscle phenotype | RMST, months | Difference in RMST vs Normal, months | 95% CI |
| --- | --- | --- | --- | --- |
| 12 | Normal | 9.67 | 0.00 | Reference |
| 12 | SMI-low | 11.55 | 1.88 | 0.76–3.04 |
| 12 | HGS-low | 6.29 | −3.39 | −4.71–−1.92 |
| 12 | Both-low | 7.51 | −2.16 | −3.81–−0.46 |
| 18 | Normal | 13.14 | 0.00 | Reference |
| 18 | SMI-low | 15.75 | 2.61 | 0.00–4.91 |
| 18 | HGS-low | 7.51 | −5.63 | −7.81–−3.32 |
| 18 | Both-low | 9.62 | −3.51 | −6.15–−0.82 |
| 24 | Normal | 16.09 | 0.00 | Reference |
| 24 | SMI-low | 18.52 | 2.43 | −1.98–6.41 |
| 24 | HGS-low | 8.52 | −7.57 | −10.55–−4.33 |
| 24 | Both-low | 11.70 | −4.39 | −8.25–−0.47 |

RMST was estimated at prespecified truncation times of 12, 18, and 24 months. Differences were calculated as the RMST for each phenotype minus that for Normal. The 95% CIs were estimated using 1,000 stratified bootstrap resamples. Normal, normal HGS and normal SMI; SMI-low, low SMI with normal HGS; HGS-low, low HGS with normal SMI; Both-low, low HGS and low SMI. CI, confidence interval; HGS, handgrip strength; RMST, restricted mean survival time; SMI, skeletal muscle index.

Table S6. Restricted mean overall survival and differences versus the Normal phenotype at 12, 18, and 24 months

| Time horizon, months | Muscle phenotype | RMST, months | Difference in RMST vs Normal, months | 95% CI |
| --- | --- | --- | --- | --- |
| 12 | Normal | 11.16 | 0.00 | Reference |
| 12 | SMI-low | 11.55 | 0.39 | −0.71–1.24 |
| 12 | HGS-low | 8.10 | −3.07 | −4.29–−1.79 |
| 12 | Both-low | 7.81 | −3.35 | −4.78–−1.85 |
| 18 | Normal | 15.49 | 0.00 | Reference |
| 18 | SMI-low | 15.87 | 0.38 | −1.77–2.49 |
| 18 | HGS-low | 9.88 | −5.61 | −7.64–−3.44 |
| 18 | Both-low | 10.20 | −5.29 | −7.97–−2.71 |
| 24 | Normal | 18.91 | 0.00 | Reference |
| 24 | SMI-low | 19.27 | 0.36 | −3.59–3.99 |
| 24 | HGS-low | 10.99 | −7.92 | −10.47–−5.32 |
| 24 | Both-low | 12.40 | −6.50 | −10.28–−2.82 |

RMST was estimated at prespecified truncation times of 12, 18, and 24 months. Differences were calculated as the RMST for each phenotype minus that for Normal. The 95% CIs were estimated using 1,000 stratified bootstrap resamples. Normal, normal HGS and normal SMI; SMI-low, low SMI with normal HGS; HGS-low, low HGS with normal SMI; Both-low, low HGS and low SMI. CI, confidence interval; HGS, handgrip strength; RMST, restricted mean survival time; SMI, skeletal muscle index.

Table S7. Sensitivity analyses of HGS and SMI modeled as continuous variables

| Outcome | Exposure | Adjusted HR (95% CI) | *P* value | Patients, n | Events, n |
| --- | --- | --- | --- | --- | --- |
| PFS | HGS, per 1-SD decrease | 2.00 (1.52–2.62) | <0.001 | 154 | 97 |
| PFS | SMI, per 1-SD decrease | 1.13 (0.92–1.39) | 0.241 | 154 | 97 |
| OS | HGS, per 1-SD decrease | 2.48 (1.85–3.32) | <0.001 | 154 | 93 |
| OS | SMI, per 1-SD decrease | 1.29 (1.05–1.58) | 0.016 | 154 | 93 |

Adjusted HRs per 1-SD decrease in HGS or SMI were estimated after adjustment for the same clinical covariates as in the primary models. CI, confidence interval; HGS, handgrip strength; HR, hazard ratio; OS, overall survival; PFS, progression-free survival; SD, standard deviation; SMI, skeletal muscle index.

Table S8. Mutually adjusted associations of standardized HGS and SMI and tests of interaction

| Outcome | Model term | Adjusted HR (95% CI) | *P* value |
| --- | --- | --- | --- |
| PFS | HGS, per 1-SD decrease | 2.53 (1.81–3.55) | < 0.001 |
| PFS | SMI, per 1-SD decrease | 0.70 (0.52–0.95) | 0.024 |
| OS | HGS, per 1-SD decrease | 3.05 (2.11–4.41) | < 0.001 |
| OS | SMI, per 1-SD decrease | 0.75 (0.54–1.03) | 0.071 |
| PFS | HGS × SMI interaction | 1.07 (0.80–1.42) | 0.657 |
| OS | HGS × SMI interaction | 1.04 (0.77–1.42) | 0.786 |

HGS and SMI were mean-centered and standardized so that HRs represent the association per 1-SD decrease. Estimates for HGS and SMI were obtained from models containing both standardized measures and the prespecified clinical covariates. Interaction estimates were obtained from exploratory models additionally containing the product of standardized HGS and SMI. Likelihood-ratio tests comparing models with and without the interaction term yielded P = 0.657 for PFS and P = 0.786 for OS. CI, confidence interval; HGS, handgrip strength; HR, hazard ratio; OS, overall survival; PFS, progression-free survival; SD, standard deviation; SMI, skeletal muscle index.

Table S9. Exploratory male-only sensitivity analysis using multivariable Cox models

| Outcome | Variable | Adjusted HR | 95% CI | *P* value |
| --- | --- | --- | --- | --- |
| PFS | Normal | 1.00 | Reference | — |
| PFS | SMI-low | 0.66 | 0.15–2.89 | 0.579 |
| PFS | HGS-low | 3.00 | 1.76–5.11 | <0.001 |
| PFS | Both-low | 2.70 | 1.47–4.98 | 0.001 |
| PFS | Age, per 1-year increase | 0.99 | 0.97–1.02 | 0.621 |
| PFS | ECOG PS, per 1-point increase | 1.74 | 1.06–2.85 | 0.028 |
| PFS | Child–Pugh class, B vs A | 1.31 | 0.75–2.29 | 0.340 |
| PFS | Maximum tumor diameter, per 1-cm increase | 1.04 | 0.99–1.10 | 0.129 |
| PFS | Tumor number, multiple vs solitary | 2.76 | 1.56–4.89 | <0.001 |
| PFS | PVTT, present vs absent | 1.20 | 0.75–1.92 | 0.438 |
| PFS | Extrahepatic spread, present vs absent | 1.38 | 0.77–2.48 | 0.274 |
| PFS | Previous HCC treatment, yes vs no | 1.85 | 1.10–3.12 | 0.020 |
| OS | Normal | 1.00 | Reference | — |
| OS | SMI-low | 0.74 | 0.17–3.30 | 0.698 |
| OS | HGS-low | 2.99 | 1.74–5.16 | <0.001 |
| OS | Both-low | 3.82 | 2.01–7.25 | <0.001 |
| OS | Age, per 1-year increase | 1.01 | 0.99–1.03 | 0.516 |
| OS | ECOG PS, per 1-point increase | 1.72 | 1.05–2.84 | 0.033 |
| OS | Child–Pugh class, B vs A | 2.02 | 1.15–3.55 | 0.014 |
| OS | Maximum tumor diameter, per 1-cm increase | 1.04 | 0.99–1.10 | 0.132 |
| OS | Tumor number, multiple vs solitary | 2.41 | 1.34–4.33 | 0.003 |
| OS | PVTT, present vs absent | 1.04 | 0.64–1.68 | 0.872 |
| OS | Extrahepatic spread, present vs absent | 1.39 | 0.77–2.51 | 0.267 |
| OS | Previous HCC treatment, yes vs no | 1.46 | 0.85–2.52 | 0.168 |

This analysis included 134 men, with 82 PFS events and 78 deaths. Models included the same covariates as the primary analyses except sex. CI, confidence interval; ECOG PS, Eastern Cooperative Oncology Group performance status; HCC, hepatocellular carcinoma; HGS, handgrip strength; HR, hazard ratio; OS, overall survival; PFS, progression-free survival; PVTT, portal vein tumor thrombosis; SMI, skeletal muscle index.

Table S10. Bootstrap optimism-corrected model performance in the AFP-inclusive parsimonious sensitivity analysis

| Model | Apparent C-index | Optimism-corrected C-index | Apparent ΔC-index | Optimism-corrected ΔC-index | Likelihood-ratio *P* value |
| --- | --- | --- | --- | --- | --- |
| Parsimonious clinical model including AFP | 0.659 | 0.630 | Reference | Reference | — |
| Parsimonious model + low SMI | 0.666 | 0.632 | 0.007 | 0.002 | 0.225 |
| Parsimonious model + low HGS | 0.725 | 0.701 | 0.067 | 0.071 | <0.001 |
| Parsimonious model + four phenotypes | 0.727 | 0.697 | 0.068 | 0.067 | <0.001 |
| Direct comparison: four phenotypes vs low HGS | — | — | — | −0.004 | 0.639 |

The parsimonious clinical model included ECOG PS, Child–Pugh class, maximum tumor diameter, tumor number, PVTT, extrahepatic spread, and AFP ≥ 400 ng/mL. Optimism-corrected estimates were obtained using 2,000 paired bootstrap resamples. The direct-comparison row compares the four-phenotype model with the model containing low HGS. AFP, alpha-fetoprotein; C-index, concordance index; ΔC-index, difference in concordance index; ECOG PS, Eastern Cooperative Oncology Group performance status; HGS, handgrip strength; PVTT, portal vein tumor thrombosis; SMI, skeletal muscle index.

Table S11. Adjusted survival analyses restricted to patients receiving lenvatinib plus an immune checkpoint inhibitor

| Outcome | Phenotype | n | Events | Adjusted HR (95% CI) | *P* value |
| --- | --- | --- | --- | --- | --- |
| PFS | Normal | 56 | 26 | 1.00 (Reference) | — |
| PFS | SMI-low | 9 | 5 | 0.86 (0.29–2.54) | 0.783 |
| PFS | HGS-low | 39 | 32 | 2.96 (1.69–5.18) | < 0.001 |
| PFS | Both-low | 23 | 13 | 2.68 (1.31–5.50) | 0.007 |
| OS | Normal | 56 | 23 | 1.00 (Reference) | — |
| OS | SMI-low | 9 | 5 | 0.91 (0.29–2.86) | 0.871 |
| OS | HGS-low | 39 | 31 | 3.47 (1.95–6.17) | < 0.001 |
| OS | Both-low | 23 | 13 | 3.78 (1.77–8.08) | < 0.001 |

Models were adjusted for age, sex, ECOG PS, Child–Pugh class, maximum tumor diameter, tumor number, PVTT, extrahepatic spread, and previous HCC treatment. Normal was the reference. CI, confidence interval; ECOG PS, Eastern Cooperative Oncology Group performance status; HCC, hepatocellular carcinoma; HGS, handgrip strength; HR, hazard ratio; ICI, immune checkpoint inhibitor; OS, overall survival; PFS, progression-free survival; PVTT, portal vein tumor thrombosis; SMI, skeletal muscle index.
